# Trends in Demographics of Neurology House Staff in the United States

**DOI:** 10.7759/cureus.17754

**Published:** 2021-09-06

**Authors:** Carolina Gil Tommee, Krishna Nalleballe, Vasuki Dandu, Vaishali Thombre, Nidhi Kapoor, Lalasa Doppalapudi, Sen Sheng, Sukanthi Kovvuru, Mitesh Lotia, Karthika Durga Veerapaneni

**Affiliations:** 1 Neurology, University of Arkansas for Medical Sciences, Little Rock, USA; 2 Neurology/Stroke, University of Arkansas for Medical Sciences, Little Rock, USA; 3 Neurology, Baptist Health Medical Center, Little Rock, USA; 4 Biostatistics and Epidemiology, University of Arkansas for Medical Sciences, Little Rock, USA; 5 Medicine, University of Arkansas for Medical Sciences, Little Rock, USA

**Keywords:** demographics, neurology, house staff, residency, fellowship

## Abstract

Background

The lack of an adequate number of neurologists is a worldwide problem. As populations age, the prevalence of neurological disorders will likely increase, thereby increasing the demand for neurologists. In addition to the growing demand, inadequate diversity in the neurology healthcare workforce still exists. The purpose of this study is to examine the demographic characteristics of neurology residents and fellows.

Methodology

This cross-sectional study used data from the following publicly available databases: Accreditation Council for Graduate Medical Education, Association of American Medical Colleges, and the United States Census Bureau. Trends (from 2007 to 2018) in demographic characteristics were assessed using the slope and the associated p-value of a simple linear regression model, with the year as the independent variable. All p-values of <0.05 were considered significant.

Results

From 2007 through 2018, the percentage of US medical school graduates in neurology residency decreased from 58% to 55% (slope = -0.25; p = 0.0004), while the percentage of international medical graduates (IMGs) decreased from 36% to 32% (slope = -0.29; p = 0.0141) and doctor of osteopathy (DO) graduates increased from 6% to 13% (slope = 0.58; p < 0.0001). Although the percentage of female neurology residents increased from 39.5% in 2007 to 43.1% in 2018 (slope = 0.03; p = 0.8659), female physicians were underrepresented in vascular neurology fellowship (34% in 2018). Collectively, the percentage of underrepresented minorities in neurology residencies was low and increased only slightly over time (from 8% in 2011 to 9% in 2018; slope = 0.17; p = 0.0788). In 2018, the proportion of underrepresented minorities was smaller in neurology fellowships (5.5% neurophysiology, 12.5% epilepsy, 10.4% neuromuscular, and 12.4% vascular) compared to the population as a whole (31.3%).

Conclusions

IMGs still play an important role in filling a significant portion of the neurology residencies and fellowships. DO graduates have slowly increased in neurology residencies and fellowships. Members of several racial/ethnic minority groups and women are underrepresented in neurology house staff and efforts need to be taken to increase diversity.

## Introduction

Both nationally and globally, there is a shortage of neurologists. With an increasing incidence of neurological disorders, this shortage is expected to worsen by 2025 [1]. In a recent survey of US allopathic school graduates, only 2.8% indicated an intent to enter a neurology residency out of 51,816 participants [2]. This decline in the national interest threatens to further dent the projected number of neurologists despite the steady rise in the number of neurology residency positions. An effort should be made to attract more medical students to the field of neurology, train non-neurologist physicians (primary care physicians), use new technology to make neurology more efficient, and utilize supervised nonphysician providers such as physician assistants and nurse practitioners. While attempts are made to improve the national interest in neurology, the void remains to be filled.

In addition, quality is not reflected by the sheer number of providers, and a diverse healthcare workforce is equally important to minimize disparities in healthcare [3]. Racial and ethnic minority providers are more likely to practice in underserved communities [4]. Patients report more satisfaction with their care and their ability to communicate with physicians from the same cultural background [5], and healthcare provider diversity has been linked to better patient outcomes in primary care [6]. Therefore, in this study, we attempt to examine the demographic characteristics of neurology trainees in the United States.

## Materials and methods

In this study, we report the demographic information of residents and fellows from 2007 to 2018 accessed from the Accreditation Council for Graduate Medical Education (ACGME) Data Resource Book [7]. This information is updated annually using the Accreditation Data System. Physician workforce diversity data were accessed through the Association of American Medical Colleges (AAMC), and US population diversity data were gathered from the US Census Bureau.

The demographic information was categorized as medical school, gender, age, and ethnicity. For graduates, according to their medical school, we divided the data of neurology residency and its subspecialties into the following three subcategories for analysis: US medical school graduates (USGs), international medical graduates (IMGs), and doctor of osteopathy (DO) graduates. For gender, the percentages of female residents/fellows were gathered and analyzed for trends in neurology and its subspecialties. For ethnicity, the percentages of Asian/Pacific Islander and underrepresented minority (URM) were calculated and analyzed in neurology and its subspecialties.

To determine who qualifies as a URM, we used the AAMC definition, “Underrepresented in medicine means those racial and ethnic populations that are underrepresented in the medical profession relative to their numbers in the general population and it consisted of African American, Mexican Americans, Native Americans (that is, American Indians, Alaska Natives, and Native Hawaiians), and mainland Puerto Ricans.”

For the total number of residencies, the following specialties were included: anesthesiology, dermatology, emergency, family medicine, internal medicine, medical genetics and genomics, neurological surgery, child neurology, nuclear medicine, obstetrics and gynecology, ophthalmology, orthopedic surgery, osteopathic neuromusculoskeletal medicine, otolaryngology, pathology, pediatrics, physical medicine and rehabilitation, plastic surgery and integrated, preventive medicine, psychiatry, radiation oncology, radiology, interventional radiology, vascular surgery, thoracic surgery, urology, and internal medicine/pediatrics.

The following neurology subspecialties were included in our study: neurophysiology, neuromuscular, epilepsy, and vascular neurology. These subspecialties were selected to represent the demographic features in our study as they represent 94% of the total number of applicants.

Trends over time (2007 to 2018) in demographic characteristics were assessed using the slope and the associated p-value of a simple linear regression model, with the year as the independent variable. All p-values of <0.05 were considered significant. Statistical analyses were conducted using SAS version 9.4 (SAS Institute, Cary, NC).

## Results

Medical school

**Figure 1:**
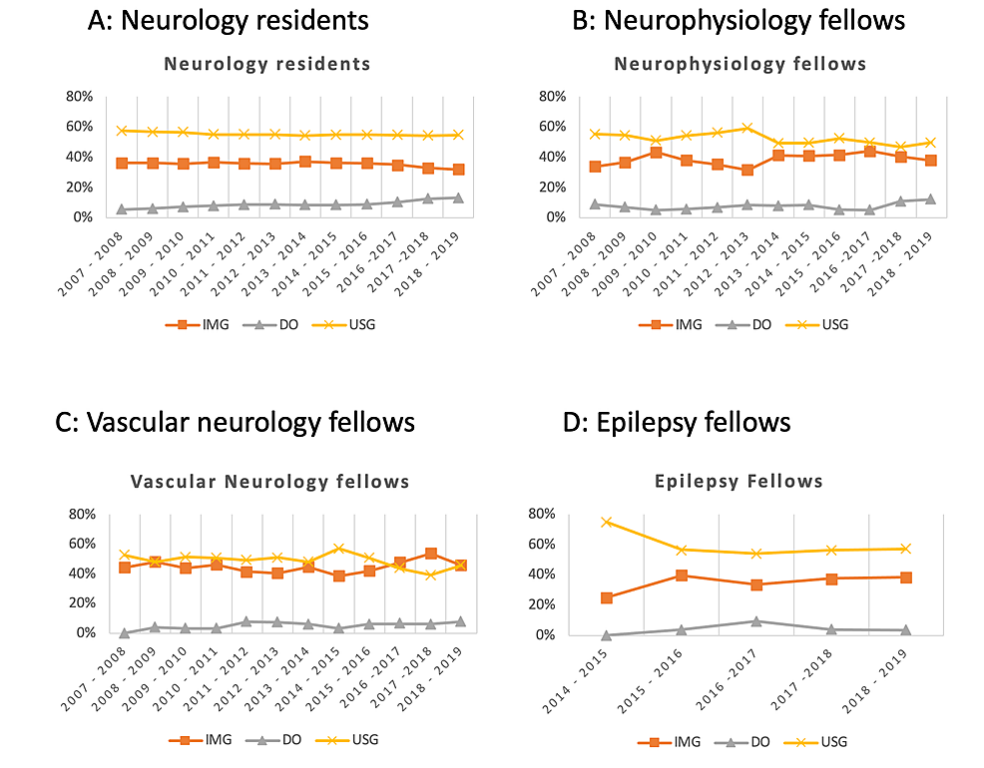
Percentages of neurology residents (A) and fellows (B, C, D) by medical school type. IMG: international medical graduates; DO: doctor of osteopathy; USG: US medical graduates

From 2007 through 2018, the percentage of neurology residents slightly decreased for USGs (58% to 55%) and IMGs (36% to 32%) but increased for DO graduates (6% to 13%) (Figure 1). For the total number of active residents, there was a slight decrease in the percentage of total USGs (65.14% to 60.98%) and IMGs (27.51% to 23.28%) from 2009 to 2018, while for DO graduates there was an increase (7.10% to 15.62%).

Between 2007 and 2019, there was an 11.85% annual increase in the number of neurology positions compared to 4.46% for internal medicine positions. There was a 10.75% annual increase in the number of US seniors that matched in neurology, while there was only a 1.91% increase in internal medicine. For neurology positions, there was a 24.85% and 24.99% annual increase in US IMGs and non-US IMGs, respectively. For internal medicine, there was a 9.00% and 4.02% annual increase in the number of positions.

In neurophysiology fellowships, the percentages of USGs, IMGs, and DO graduates ranged from 47% to 59%, 32% to 44%, and 5% to 12%, respectively, with trends similar to residencies. In neuromuscular fellowships, the percentages of USGs, IMGs, and DO graduates ranged from 39% to 62%, 28% to 47%, and 0% to 11%, respectively. From 2007 through 2017, the percentages of USGs in vascular neurology fellowships decreased from 53% to 39%, but the percentages of IMGs increased from 44% to 54%. However, in 2018, IMGs and USGs had the same percentage (46%) as DO graduates at 8%. From 2014 through 2018, the percentages of USGs in epilepsy decreased from 75% to 57%, whereas the percentages of IMGs in epilepsy increased from 25% to 38%. The highest percentage of DO graduates in epilepsy was observed in 2016 (9%), which decreased by 2018 (4%) (Figure 1).

Gender

**Figure 2:**
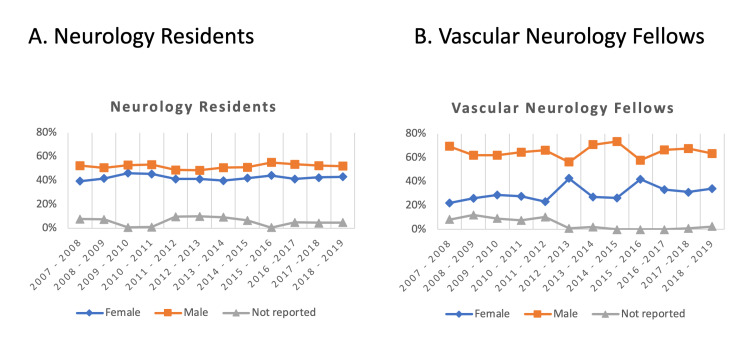
Percentages of neurology residents (A) and vascular neurology fellows (B) by gender.

From 2007 through 2018, the percentage of female neurology residents increased (39.5% to 43.1%) (Figure 2). The trend of female neurology residents was similar to the total active residents (46% vs. 42% in 2009-2010 and 43% vs. 44% in 2018-2019). The percentage of female fellows slowly increased over the years. In 2018, the percentage of female neurology residents and fellows was lower than that of the overall US population (43.10% neurology, 41.72% neurophysiology, 38.81% neuromuscular, and 34.11% vascular vs. 50.80% overall), except in epilepsy fellowships (54.46%) (Table 1). Neurology residencies and fellowships had a higher female percentage than in the overall physician population (43.10% neurology, 41.72% neurophysiology, 54.46% epilepsy, and 38.81% neuromuscular vs. 35.80% overall), except in vascular fellowship (34.11%).

**Table 1:** Percentages of neurology house staff by gender in 2018.

Characteristic	Men	Women	Not reported
US population	49.20%	50.80%	0.00%
Total active physicians	64.10%	35.80%	0.20%
Neurology residents	52.06%	43.10%	4.84%
Neurophysiology fellowship	57.06%	41.72%	1.23%
Epilepsy fellowship	44.64%	54.46%	1.00%
Neuromuscular fellowship	59.70%	38.81%	1.49%
Vascular fellowship	63.57%	34.11%	2.33%

Age

The average age of a first-year neurology resident was 30.35 (range: 30.1-30.6), while for the specialties combined the average age was 34.17 (range: 33.86-34.46).

Ethnicity

From 2011 to 2018, the percentage of residents/fellows of URM remained similar, with the exception of the percentage of Hispanic fellows which increased from 0% in 2011 to 8% in 2018. In 2018, the percentage of residents and fellows of Asian or Pacific Islander origin was higher than that of the overall US population (20.32% neurology, 24.5% neurophysiology, 16% epilepsy, 9% neuromuscular, and 22% vascular vs. 5.8% overall). Compared to total active physicians, the percentage among residents and fellows of Asian or Pacific Islander origin was similar or even higher, except in neuromuscular fellowship (9% vs. 17.2%).

In 2011-2012, the total percentage of Asian residents was 16.58% vs. 19.04% for neurology residents, while the total percentage of URM was 9.67% vs. 8.08% for neurology residents. In 2018-2019, the total percentage of Asian residents was 16.70% vs. 20.32% for neurology residents. The percentage of URM was 9.97% for total residents vs. 9.02% for neurology residents.

Collectively, the percentage of URM residents and fellows in 2018 was lower compared to the overall US population (9% neurology, 5.5% neurophysiology, 12.5% epilepsy, 10% neuromuscular, and 12% vascular vs. 31.3% overall). Even when compared to the percentage of URM in the active physician population (11%), the percentages of URM were lower in neurology residencies (9%) and neurophysiology (5.5%) and neuromuscular (10%) fellowships. The percentage of URM was higher in epilepsy (12.5%) and vascular neurology (12%) fellowships. The percentage of URM in neurology residencies increased only slightly over time (from 8% in 2011 to 9% in 2018).

## Discussion

This analysis highlights that the field of neurology has a higher percentage of IMGs than other specialties. According to ACGME (2018), neurology residency was ranked fourth in the percentage of IMGs (32%), preceded by nuclear medicine (70.7%), pathology (45.2%), and internal medicine (38.9%). The high percentage of IMGs seen in neurology could be a result of USGs opting for specialties with a better lifestyle, remuneration, and less burnout [8]. Whether IMG specialization is a personal choice or merely a reflection of gap-filling after US graduates have chosen, the neurology workforce is still heavily dependent on IMGs.

The trend of neurology residency by medical school type has had a similar trend compared to the total active residents. In general, the number of neurology positions, as well as the number of residents matching every year (US senior, US IMGs, non-IMGs) has increased significantly compared to other specialties such as internal medicine.

Even though the percentage of female neurology residents increased during the study period, neurology was 14th out of the 34 specialties for the percentage of female physicians. Obstetrics and gynecology had the most female residents, while surgical specialties had the least. The trend of female neurology residents was similar to the total active residents. Interestingly, in the neurophysiology, neuromuscular, and epilepsy subspecialties, women outnumbered men in 2017, while vascular fellowship is still predominantly male. Female neurologists tend to work about 14% fewer hours in direct patient care compared to their male counterparts [1]. This could be attributed to a greater degree of part-time status, childbearing responsibilities, and relatively earlier attrition from patient care [8], factors that could impact the choice of subspecialty.

The field of neurology continues to be underrepresented by minorities. Compared to the total number of all residencies, we found that neurology has a similar trend. We found lower percentages of URM in neurology residencies and neurophysiology and neuromuscular fellowships compared to active physicians overall. URM underrepresentation is driven by multiple factors, including racial, gender, and ethnic hiring biases [9], as well as lack of institutional commitment [10]. Studies have shown that increasing diversity in healthcare professionals helps reduce the racial/ethnic disparities in healthcare delivery [11]. Identifying barriers that hinder workforce diversity is just as essential for improving the healthcare of URMs and the overall population. Each minority possesses unique academic and social backgrounds that add to the diversity of healthcare professionals.

Our study also showed the percentages of individuals of Asian/Pacific Islander origin among neurology residency (20.32%) and neurology subspecialties (16% to 24.5%, except neuromuscular, 9%) are higher than that in the US population and the overall physician population. Several retrospective studies have shown that the percentage of individuals of Asian/Pacific Islander origin varies among other specialties such as psychiatry (10% to 16.7%), orthopedics (10.73% to 13.14%), and internal medicine (23.7% to 28.7%) [12,13], but has remained stable in individual specialties over a period of 10 years. This phenomenon was also observed in neurology with a percentage of at least 20% over a period of 10 years. The exact explanation for the above phenomenon is still unclear, although different recruitment efforts are applied and analyzed in other specialties [13]. This should be investigated in future studies and applied to other racial/ethnic minorities as well.

Limitations

Neurology residency is a four-year program where the first year is completed in either preliminary or transitional medicine. While most programs match candidates for the four-year program, some candidates join in the second year after finishing preliminary or transitional medicine in a different program. The demographics of these individuals might not be represented in the database. Further, gender and race/ethnicity data were missing for some physicians. Another limitation of our study was the limited number of fellowships included in it. While we understand that there are other subspecialties available, the data found were limited for inclusion in this study.

## Conclusions

This study provides useful insights into the demographics of neurology trainees. IMGs continue to remain essential to the neurology workforce by filling an insignificant number of residency and fellowship positions, and the number of DO candidates has steadily increased as well. This information becomes more important as the interest in neurology among USGs has declined recently. Female and URM populations remain underrepresented, and efforts to increase diversity among the neurology healthcare workforce are needed. Further studies would need to be performed over time to follow the trends as well as to understand the career choice differences among IMGs, URM, and women in the choice of specialty.
